# Supplementary material for: Did people really drink bleach to prevent COVID-19? A guide for protecting survey data against problematic respondents
Source: PLoS One. 2023 Jul 5;18(7):e0287837. doi: 10.1371/journal.pone.0287837 (PMC10321604; doi:10.1371/journal.pone.0287837)

PLOS ONE

Did People Really Drink Bleach to Prevent COVID-19? A Tale of Problematic Respondents and A Guide for Measuring Rare Events in Survey Data

--Manuscript Draft--

**Manuscript Number:**	PONE-D-22-20912
**Article Type:**	Research Article
**Full Title:**	Did People Really Drink Bleach to Prevent COVID-19? A Tale of Problematic Respondents and A Guide for Measuring Rare Events in Survey Data
**Short Title:**	BLEACH CONSUMPTION DURING COVID
**Corresponding Author:**	Rachel Hartman<br>University of North Carolina at Chapel Hill<br>Chapel Hill, North Carolina UNITED STATES
**Keywords:**	Public health; COVID-19; survey methodology; Data Quality
**Abstract:**	Society is becoming increasingly dependent on survey research. However, survey respondents who are non-attentive, respond randomly to survey questions, and misrepresent who they are and their true attitudes can have a large impact on the survey outcomes. However, the impact that such respondents can have on public health research has rarely been systematically examined. In this study, we attempt to replicate a CDC study suggesting that people engaged in highly dangerous cleaning practices during the Covid-19 pandemic, including ingesting household cleaners such as bleach. Across two studies (N = 1,288), we show that 80-90% of reported ingestion of household cleaners are made by problematic respondents. These respondents report impossible claims such as “recently having had a fatal heart attack” at a similar rate to ingesting household cleaners. Additionally, respondents’ frequent misreading or misinterpreting the intent of questions accounted for the remaining claims. Once inattentive, acquiescent, and careless respondents are taken out of the analytic sample we find no evidence that people ingested cleaning products to prevent COVID-19 infections. The relationship between dangerous cleaning practices and health outcomes also becomes non-significant once problematic respondents are taken out of the analytic sample. These results show that reported ingestion of household cleaners and other similar dangerous practices are an artifact of problematic respondent bias. These findings have important implications for public health and medical survey research, as well as for best practices for avoiding problematic respondents in surveys.
**Order of Authors:**	Leib Litman, PhD
	Zohn Rosen, PhD
	Cheskie Rosenzweig
	Sarah L Weinberger-Litman, PhD
	Aaron J Moss, PhD
	Rachel Hartman
	Jonathan Robinson, PhD
**Additional Information:**	
**Question**	**Response**
**Financial Disclosure**<br><br>Enter a financial disclosure statement that describes the sources of funding for the work included in this submission. Review the submission guidelines for detailed requirements. View published research articles from *PLOS ONE* for specific	The author(s) received no specific funding for this work.

Powered by Editorial Manager® and ProduXion Manager® from Aries Systems Corporation

examples.<br><br>This statement is required for submission and **will appear in the published article** if the submission is accepted. Please make sure it is accurate.<br><br>**Unfunded studies**<br>Enter: *The author(s) received no specific funding for this work.*<br><br>**Funded studies**<br>Enter a statement with the following details:<br>• Initials of the authors who received each award<br>• Grant numbers awarded to each author<br>• The full name of each funder<br>• URL of each funder website<br>• Did the sponsors or funders play any role in the study design, data collection and analysis, decision to publish, or preparation of the manuscript?<br>• **NO** - Include this sentence at the end of your statement: *The funders had no role in study design, data collection and analysis, decision to publish, or preparation of the manuscript.*<br>• **YES** - Specify the role(s) played.<br><br>* typeset	
**Competing Interests**<br><br>Use the instructions below to enter a competing interest statement for this submission. On behalf of all authors, disclose any competing interests that could be perceived to bias this work—acknowledging all financial support and any other relevant financial or non-financial competing interests.<br><br>This statement is required for submission and **will appear in the published article** if the submission is accepted. Please make sure it is accurate and that any funding sources listed in your Funding Information later in the submission form are also declared in your Financial Disclosure statement.	The authors have declared that no competing interests exist.

Powered by Editorial Manager® and ProduXion Manager® from Aries Systems Corporation

View published research articles from *PLOS ONE* for specific examples.<br><br>**NO authors have competing interests**<br><br>Enter: *The authors have declared that no competing interests exist.*<br><br>**Authors with competing interests**<br><br>Enter competing interest details beginning with this statement:<br><br>*I have read the journal's policy and the authors of this manuscript have the following competing interests: [insert competing interests here]*<br><br>* typeset	
**Ethics Statement**<br><br>Enter an ethics statement for this submission. This statement is required if the study involved:<br><br>• Human participants<br>• Human specimens or tissue<br>• Vertebrate animals or cephalopods<br>• Vertebrate embryos or tissues<br>• Field research<br><br>Write "N/A" if the submission does not require an ethics statement.<br><br>General guidance is provided below. Consult the submission guidelines for detailed instructions. **Make sure that all information entered here is included in the Methods section of the manuscript.**	The study was exempt from review because it is an anonymous survey. Exempt status was confirmed by InterReview IRB. The consent process was embedded within the survey (participants read a consent form that said at the end "by proceeding with the survey you're providing your consent. If you do not consent, you may exit the survey now"

**Format for specific study types**<br><br>**Human Subject Research (involving human participants and/or tissue)**<br>• Give the name of the institutional review board or ethics committee that approved the study<br>• Include the approval number and/or a statement indicating approval of this research<br>• Indicate the form of consent obtained (written/oral) or the reason that consent was not obtained (e.g. the data were analyzed anonymously)<br><br>**Animal Research (involving vertebrate animals, embryos or tissues)**<br>• Provide the name of the Institutional Animal Care and Use Committee (IACUC) or other relevant ethics board that reviewed the study protocol, and indicate whether they approved this research or granted a formal waiver of ethical approval<br>• Include an approval number if one was obtained<br>• If the study involved *non-human primates*, add *additional details* about animal welfare and steps taken to ameliorate suffering<br>• If anesthesia, euthanasia, or any kind of animal sacrifice is part of the study, include briefly which substances and/or methods were applied<br><br>**Field Research**<br><br>Include the following details if this study involves the collection of plant, animal, or other materials from a natural setting:<br>• Field permit number<br>• Name of the institution or relevant body that granted permission	
**Data Availability**<br><br>Authors are required to make all data underlying the findings described fully available, without restriction, and from the time of publication. PLOS allows rare exceptions to address legal and ethical concerns. See the PLOS Data Policy and FAQ for detailed information.	Yes - all data are fully available without restriction

A Data Availability Statement describing where the data can be found is required at submission. Your answers to this question constitute the Data Availability Statement and **will be published in the article**, if accepted.<br><br>**Important:** Stating 'data available on request from the author' is not sufficient. If your data are only available upon request, select 'No' for the first question and explain your exceptional situation in the text box.<br><br>Do the authors confirm that all data underlying the findings described in their manuscript are fully available without restriction?	
**Describe where the data may be found in full sentences. If you are copying our sample text, replace any instances of XXX with the appropriate details.**<br><br>• If the data are **held or will be held in a public repository**, include URLs, accession numbers or DOIs. If this information will only be available after acceptance, indicate this by ticking the box below. For example: *All XXX files are available from the XXX database (accession number(s) XXX, XXX.).*<br>• If the data are all contained **within the manuscript and/or Supporting Information files**, enter the following: *All relevant data are within the manuscript and its Supporting Information files.*<br>• If neither of these applies but you are able to provide **details of access elsewhere**, with or without limitations, please do so. For example:<br><br>*Data cannot be shared publicly because of [XXX]. Data are available from the XXX Institutional Data Access / Ethics Committee (contact via XXX) for researchers who meet the criteria for access to confidential data.*<br><br>*The data underlying the results presented in the study are available from (include the name of the third party*	All data files are available on OSF at https://osf.io/fzx9v/?view_only=90d2039f61384f9b9dd99b72ca547c9a

and contact information or URL).<br>• This text is appropriate if the data are owned by a third party and authors do not have permission to share the data.<br><br>* typeset	
Additional data availability information:	

Manuscript

Click here to access/download;Manuscript;Did people really drink bleach_Revision.full.docx

Did People Really Drink Bleach to Prevent COVID-19? A Tale of Problematic Respondents and A Guide for Measuring Rare Events in Survey Data

Leib Litman[1,2], Zohn Rosen[3], Cheskie Rosenzweig[1,4], Sarah L. Weinberger-Litman[5], Aaron J. Moss[1], Rachel Hartman[1,6] and Jonathan Robinson[1,7]

[1]CloudResearch, Queens, NY

[2]Department of Psychology, Lander College

[3]Department of Health Policy and Management, Mailman School of Public Health, Columbia University

[4]Department of Clinical Psychology, Columbia University

[5]Department of Psychology, Marymount Manhattan College

[6]Department of Psychology & Neuroscience, University of North Carolina at Chapel Hill

[7]Department of Computer Science, Lander College

Author Note

Leib Litman: https://orcid.org/0000-0001-6598-2701
Zohn Rosen: https://orcid.org/0000-0001-7758-9739
Cheskie Rosenzweig: https://orcid.org/0000-0003-2403-6940
Sarah Weinberger-Litman: https://orcid.org/0000-0002-0893-8445
Aaron J. Moss: https://orcid.org/0000-0003-4396-4128
Rachel Hartman: https://orcid.org/0000-0001-9714-3224

Correspondence concerning this article should be addressed to Leib Litman, leib.litman@touro.edu

Abstract

Society is becoming increasingly dependent on survey research. However, survey respondents who are non-attentive, respond randomly to survey questions, and misrepresent who they are and their true attitudes can have a large impact on the survey outcomes. However, the impact that such respondents can have on public health research has rarely been systematically examined. In this study, we attempt to replicate a CDC study suggesting that people engaged in highly dangerous cleaning practices during the Covid-19 pandemic, including ingesting household cleaners such as bleach. Across two studies (N = 1,288), we show that 80-90% of reported ingestion of household cleaners are made by problematic respondents. These respondents report impossible claims such as “recently having had a fatal heart attack” at a similar rate to ingesting household cleaners. Additionally, respondents’ frequent misreading or misinterpreting the intent of questions accounted for the remaining claims. Once inattentive, acquiescent, and careless respondents are taken out of the analytic sample we find no evidence that people ingested cleaning products to prevent COVID-19 infections. The relationship between dangerous cleaning practices and health outcomes also becomes non-significant once problematic respondents are taken out of the analytic sample. These results show that reported ingestion of household cleaners and other similar dangerous practices are an artifact of problematic respondent bias. These findings have important implications for public health and medical survey research, as well as for best practices for avoiding problematic respondents in surveys.

Introduction

Surveys are one of the most common sources of data in social science[1–3], political science[4–7], public health[8,9] and medical research[10,11], informing public policy, medical practice and public opinion. Despite the widespread use of survey research, self-report data has come under increasing scrutiny over the last ten years due to data quality concerns.

One of the major threats to validity in survey research comes from participants who are inattentive[12–14], "mischievous", providing responses that are intentionally false or misleading, or "acquiescent", systematically responding "yes" to any question[15–21]. In the present research, we collectively refer to these respondents as "problematic respondents." Problematic respondents can bias the results of surveys by dramatically inflating point estimates and by creating illusory associations. In the current study we examine how problematic respondents can bias estimates of health-related behaviors. Specifically, we examine the validity of a survey conducted by the Centers for Disease Control and Prevention (CDC), which found that Americans engaged in highly dangerous practices in response to the COVID-19 pandemic, including the ingestion of bleach and household cleaner[22].

Estimates of rare events, such as the ingestion of household cleaning products, are particularly prone to problematic-respondent bias[16,23]. The goal of the present study is to examine whether the rate of reported dangerous cleaning practices, and the relationship between dangerous cleaning practices and health outcomes, were overinflated in the CDC study[22] due to problematic respondents. Another goal of this paper is to examine several different approaches to reducing problematic responses and then demonstrate how, through their proper application, data quality can be vastly improved when collecting data online.

Inflated Estimates

Evidence that problematic respondents can alter the outcomes of surveys began to accumulate as early as the 1970s. Having observed unusual patterns in survey data on self- reported illicit drug use, ~~some~~ Petzel and colleagues began to suspect that some respondents were exaggerating their drug

use in their self-reports[24]. To examine this issue, they created a paradigm for catching potentially problematic respondents which involved incorporating questions about a fictitious drug in the survey. They found that 4% of people reported using this fictitious drug, and that these people were also much more likely to report using other drugs, suggesting a general propensity toward acquiescence bias among these respondents.

A similar approach to identifying problematic respondents was used in a nationwide school-based study in Norway, in which close to 12,000 participants responded to questions about drug use[25]. The authors found that respondents who reported buying and using the fictitious drug “Zetacyclin” also reported disproportionately heavy use of other drugs such as heroin and LSD. Because heroin and LSD use is relatively rare, excluding these respondents from the analytic sample made a critical difference for inferences about the nationwide incidence of drug use.

Some of the most compelling demonstrations of the dramatic impact that problematic respondents can have on surveys come from the national Longitudinal Study of Adolescent Health (Add Health)[15]. Add Health uses multiple measurement methods, including surveys, in- person interviews and interviews with parents, which allows for survey responses to be directly cross-referenced with in-person interviews. There have been multiple instances where in-person interviews directly contradicted survey responses. For example, 20% of respondents falsely reported not being born in the US, and 19% falsely reported being adopted. These responses were later contradicted by the adolescents’ parents during in-person interviews. Further, in what is perhaps the most striking example of the potential for problematic respondents to invalidate the results of medical surveys, only 2 out of the 253 people who indicated that they had used an artificial limb for more than a year confirmed their response in a follow-up in-person interview.

Inflated Correlations

One clear pattern that emerges from studies that have examined problematic respondents is that once a respondent provides a false response, they are more likely to provide other false and problematic data. In other words, demonstrably false responses to some questions are typically a good

indication that the entire survey should be treated with suspicion and should be considered for exclusion from the analysis. For example, people who falsely reported being adoptees or having a false limb were also less likely to be consistent when providing demographic information including gender, age, and race. While the correlation between the respondents' age provided on the survey and their age determined from an at home interview was above .95 for people who did not provide false data, that correlation was only .41-.47 for people who provided false reports. Further, the participants who provided false information also endorsed extreme responses on a wide range of behaviors such as alcohol consumption, leading to inflated and illusory between-groups disparities. A reanalysis of the Add Health data after removing problematic respondents from the sample led the authors to conclude that several original reported disparities were "substantially overstated," and led to retractions of published reports[15,21].

Problematic Respondents in Online Surveys

In contrast to the Add Health dataset, most online survey responses cannot be confirmed with in-person interviews. Thus, to combat problematic responses, numerous data validity screening techniques have been developed. These screening techniques can occur either prior to the survey, preventing problematic respondents from participating[26], or they can appear within the survey, identifying problematic respondents to be excluded from the analytic sample[12,27–32].

Using these techniques, researchers have reanalyzed extant survey data while controlling for problematic respondent bias to better understand how findings in specific research areas may have been affected by problematic responses[33]. These efforts have helped to reveal that problematic respondents can drastically attenuate results, at times leading researchers to conclude that previously established findings lack validity[15,33,34]. This is especially important when the studies in question have direct implications for public health and public policy[35].

Problematic respondents are not bound to specific modalities of survey sampling, such as specific national databases or particular online survey platforms, and are not limited to specific demographic populations. Rather, problematic respondent bias is a ubiquitous problem that requires

mitigation in any type of survey[18,26,26], leading data quality researchers to call for the inclusion of rigorous methodology to support the validity of estimates drawn from survey data[36].

One increasingly popular modality of collecting survey responses is via online opt-in panels. Such panels constitute more that 80% of currently conducted public opinion polls[23], and are increasingly being used for data collection in public health, political science, and social and behavioral sciences[26,37]. A large literature on opt-in panels indicates that the percentage of problematic respondents on such panels is substantial[6,23,26,38–44]. Estimates of the magnitude of problematic respondent bias in online opt-in panel platforms vary between 4-7%[23] and 30%[26], although in some studies the magnitude of inattention has been as high as 50%[43].

The problematic survey responses obtained in online panels are not random. The largest and most comprehensive study done to date to systematically examine problematic responses in online opt-in panels shows that responses tend to be systematically skewed toward positive answer choices[23]. Specifically, this means that when provided with a yes/no response option, problematic respondents will be more likely to choose “yes” over “no”. This acquiescence bias is particularly concerning in studies that aim to measure rare events. This is because even a small percentage of respondents who falsely answer “yes” to questions about rare events will make a non-existent phenomenon appear to be real. The present study addresses this issue in the public health context. Specifically, we examine how false “yes” responses can artificially inflate estimates of rare public health-relevant behaviors in online opt-in samples. We also explore whether reported correlations between dangerous health behaviors and negative health outcomes may be inflated due to problematic responses.

Health Behaviors During COVID-19

The Covid-19 pandemic has had a profound influence on daily health-related practices in the United States and around the world. The World Health Organization (WHO) and the CDC have issued multiple behavior guidelines to help curb the spread of infection, including wearing a face mask and social distancing. Some of the most important health-related guidelines relate to cleanliness practices, including the need to wash hands thoroughly and often, and to avoid hand-to-face contact[45,46].

Previous research has shown that even before COVID-19, cleanliness and contamination concerns have led people to engage in a variety of cleaning practices to reduce the likelihood of infection, particularly surrounding food cleanliness[47,48]. At times, contamination concerns can lead people to engage in dangerous cleaning practices, such as overusing antimicrobial products that can lead to skin damage and cause other health problems[49]. It is thus reasonable to expect that during a pandemic, when fear of contamination and infection is very high, people will be even more likely to engage in a variety of cleanliness practices as they seek to protect their health.

In June 2020, a few months after the start of the COVID-19 pandemic, the CDC reported the results of a survey they conducted using an online opt-in panel[22]. The survey asked American respondents if they had engaged in several cleaning practices to prevent a COVID-19 infection during the month of April 2020. Their data revealed that 39% of Americans engaged in at least one cleaning practice not recommended by the CDC. For our purposes, we categorize these practices as either moderately or highly dangerous. Moderately dangerous practices include washing food products with bleach (19% of respondents reported engaging in this behavior), using household cleaner or disinfectant on one's skin (18%), misting the body with cleaning or alcohol spray (10%), and inhaling the vapors of household cleaners like bleach (6%). Highly dangerous practices included drinking or gargling household cleaning products (4%), drinking or gargling soapy water (4%), or drinking or gargling diluted bleach (4%) in order to prevent Covid-19 infection.

The finding that Americans were engaging in dangerous cleaning practices at such high rates is alarming. It suggests that fears of COVID-19, coupled with a lack of knowledge about the dangers of such practices, are leading tens of millions of people to engage in behaviors that can damage their overall health. Indeed, the scale at which people are engaging in such practices may be revealing an area of public health concern that requires large-scale intervention.

However, these results should be interpreted with caution, for several reasons. As in any study aiming to detect rare events using survey research, problematic responses can severely bias the estimates of such behaviors. For this reason, we sought to examine whether reports of dangerous

cleaning practices, and claims of the highly dangerous ingestion of cleaning products in particular, can be attributed in part or in whole to problematic respondent bias.

The Present Research

Our overall goal in this study was to examine whether reports of dangerous cleaning practices such as ingesting household cleaners to prevent COVID-19 infection can be detected after controlling for problematic respondent bias. Specifically, we sought to systematically measure the magnitude of problematic respondent bias in influencing estimates of dangerous cleaning practices, with a focus on the ingestion of household cleaners including bleach, soapy water, and household disinfectant. We aimed to determine what role problematic respondents may play on the reporting of these practices, and to more accurately measure how widespread such practices are. Since problematic responses may also artificially inflate correlations between measures, we also aimed to investigate whether dangerous cleaning practices remained highly associated with negative health outcomes after removing problematic respondents from the sample. Data and syntax are available at https://osf.io/fzx9v/?view_only=90d2039f61384f9b9dd99b72ca547c9a.

Study 1

Hypotheses

Hypothesis 1: We hypothesized that problematic respondents would be responsible for most reports of dangerous cleaning practices, and that this would be especially true for the three highly dangerous practices: drinking or gargling bleach, disinfectant, or household cleaner.

Hypothesis 2: We expected that dangerous behaviors with lower (vs. higher) reported frequencies would have a greater proportion of affirmative responses from problematic respondents.

Hypothesis 3: We predicted that our data quality measures would have a very low false positive rate. specifically, we expected that less than 1% of non-problematic respondents would report ingesting household cleaners.

Hypothesis 4: We hypothesized that, reflecting an acquiescence bias, there would be a significant correlation between the rates of reported ingestion of household cleaning products and other

implausible/impossible behaviors such as having experienced a fatal heart attack.

Hypothesis 5: Again reflecting an acquiescence bias, we expected the association between dangerous cleaning practices and negative health outcomes to be high among problematic respondents, and low among non-problematic respondents.

Method

Participants and Design

The current study was a replication of the CDC study[22]. Aside from the addition of data quality measures we used the identical survey design, question wording, online sample provider, and sampling methodology as reported in the CDC study. The study was exempt from review because it is an anonymous survey. Exempt status was confirmed by InterReview IRB. We collected data from a national sample of 600 respondents during the week of June 10th-June 17th, 2020. The sample was matched to the U.S. Census on gender, age, race, and region (see Table 1 for the respondents' demographics). After providing informed consent, participants responded to the measures described below.

Table 1
Basic demographics.

	**Study 1(%)**	**Study 2 (%)**
Age	50.0 (17.2)	43.1 (17.8)
**Gender**		
Female	53	59
**Race**		
White	71	67
Black	13	13
American Indian or Alaska Native	2.2	2.2
Asian	8	8
Native Hawaiian or Pacific Islander	0.2	0.8
Multiracial/Other	2.5	9
**Hispanic**		
Yes	17	23
**Highest Degree**		
No college degree	35	48
Associate degree	10	12
Bachelor's degree	31	23
Graduate degree	24	17
**Region**		
Northeast	25	24
Midwest	21	23
South	35	31
West	19	17

Materials

Cleaning Practices

The survey included questions about the cleaning behaviors respondents engaged in as a response to the COVID-19 pandemic. In addition to asking about an increase in housecleaning frequency (which would not be considered dangerous), the moderately dangerous practices included washing produce with bleach, using household cleaner to clean or disinfect one's skin, misting the

body with cleaning or alcohol spray, inhaling the vapors of household cleaners, and the highly dangerous practices included drinking or gargling household cleaner, soapy water, or diluted bleach.

Negative Health Outcomes

Participants indicated whether they had experienced any of a list of health effects due to using cleaners or disinfectants in the past month. These include nose or sinus irritation, skin irritation, eye irritation, dizziness, lightheadedness, or headache, upset stomach or nausea, and breathing problems.

Data Quality Measures

We employed a combination of instruments to address multiple known characteristics of problematic respondent bias.

Attentiveness and English Language Comprehension. We adapted a procedure developed by Chandler and colleagues[26] which checks for attentiveness and basic English language comprehension by presenting participants with a target word and asking them which of four other words is most related to the target word. For example, participants may see a target word, “Fruit” and would need to select the most related word from a list: “Table,” “Medicine,” “Pencil,” or “Banana.” Chandler et al. showed that this instrument identified the vast majority of inattentive respondents in online samples, while having very low levels of false positives (i.e., almost all non-problematic respondents answer these questions correctly).

Here, we improved on Chandler et al.’s methods by having refined and extensively tested each question in the instrument. The stimuli were generated by an associative semantic network algorithm, which assigned weights to word-pairs based on corpora of English language texts. Word-pairs were assigned weights based on the similarity and frequency with which they appear together. Of the four response options, the correct response was highly associated with the target (e.g., Fruit-Banana) and the other three had low associations with the target (e.g., Fruit-Pencil). Only very common words that have a high frequency of occurrence in the English language were used as targets or response options, to avoid education bias. Screening questions with a pass rate of 95% or above were used, as based on pilot testing conducted on independent participant samples. Using this approach, we developed a

library of questions so that different combinations of screening questions can be presented to different participants. This prevents bots and problematic respondents from learning correct responses to these questions, sharing them online, and creating scripts for response automation.

In addition to this measure, we also included two additional questions in the survey. These questions were (1) The trophy doesn't fit into the brown suitcase because it is too large. What is too large?" and (2) "Have you ever used the internet?". Each of these had several response options, with only one correct answer ("The trophy" and "Yes", respectively).

Acquiescence. The second method we used was originally developed by Petzel and colleagues[24] and subsequently used by multiple other methodologists[25,26,33]. It involves incorporating questions within the survey about highly unlikely or impossible behaviors such as using a non-existent drug or 'eating concrete for its iron content' to identify problematic respondents. We asked respondents three questions where the only plausible answer is "No." Specifically, we asked (1) "Do you know what the word wuttlet means?" (2) "Have you ever suffered a fatal heart attack?" and (3) "From memory, can you recall the name of every senator who has ever served in the U.S. Senate?" Each of these questions was pre-tested to ensure that over 95% of attentive respondents answer in the expected way.

Results

We first classified respondents into one of two groups: "problematic respondents" were those who responded incorrectly to any of our data quality measures, and "non-problematic" respondents were those who responded correctly to all data quality measures. Overall, 460 respondents (76.7%) were "non-problematic," and 140 respondents (23.3%) were problematic.

Who Reports Engaging in Dangerous Cleaning Practices?

Across the full sample, for the moderately dangerous behaviors, we found that 12.4% of respondents reported washing produce with bleach, 15.9% reported using household cleaner to clean or disinfect one's skin, 12.1% reported misting the body with cleaning or alcohol spray, and 5.8% reported inhaling the vapors of household cleaners. When it came to the highly dangerous behaviors,

we found that, 4% reported drinking or gargling soapy water, and 4.7% reported drinking or gargling household cleaner, and 3.8% of respondents reported drinking or gargling diluted bleach solution. Especially with regard to the highly dangerous practices, these results mirror the CDC's findings[22].

To address H1, we examined the reports of engaging in dangerous cleaning practices among problematic vs. non-problematic respondents. As predicted, problematic respondents provided the vast majority of the affirmative responses to questions about dangerous cleaning behaviors, particularly for the highly dangerous behaviors (see Figure 1). For each of the dangerous cleaning practices, the likelihood of a "yes" response was between 300% and 2400% higher among problematic respondents vs. non-problematic responses. Equally as important, the opposite pattern was observed for increases in typical non-dangerous cleaning practices. It is expected that normal cleaning behaviors would increase during the pandemic driven by public health recommendations. Problematic respondents under-reported increases in normal cleaning behavior by close to 20%. Thus, problematic respondents severely overreport high-risk cleaning practices and underreport regular cleaning practices.

FIGURE 1. Comparisons of problematic and non-problematic respondents' reports of cleaning and disinfection practices between April and June, 2020.

Confirming H2, we found that dangerous behaviors with lower (vs. higher) reported frequencies had a greater proportion of affirmative responses from problematic respondents. The lowest discrepancy between problematic and non-problematic respondents was in the moderately dangerous behavior people most frequently engaged in—using cleaner or disinfectant on one's hands or skin. For this behavior, problematic respondents were approximately three times as likely as non-problematic respondents to respond affirmatively (37.9% vs. 12.2%). On the other hand, the highest discrepancy between problematic and non-problematic respondents was observed for the least common highly dangerous behaviors: household cleaner ingestion (i.e., drinking/gargling household cleaner, soapy water, or diluted bleach). Overall, 31.4% of problematic respondents reported engaging in at least one of these practices. In comparison, only 1.2% of non-problematic respondents reported

engaging in at least one of these cleaning practices.

Our third hypothesis (H3) was that our data quality measures would have a very low false positive rate. Specifically, we expected that less than 1% of non-problematic respondents would report ingesting household cleaners. As can be seen in Figure 1, the percentages of non-problematic respondents who reported ingesting household cleaner, soapy water, and diluted bleach were .9%, .6%, and .5%. Overall, 1.2% of non-problematic respondents reported ingesting any of these three products. Looked at another way, 88% of those who reported drinking or gargling household cleaner, soapy water, or diluted bleach, were identified as problematic respondents (see Figure 2).

FIGURE 2. Probability of being labeled a problematic respondent based on whether participants reported ingesting a household cleaner.

Acquiescence Bias

We found that 11.9% of respondents reported knowing what the word "wuttlet" means, despite it being a fake word. Further, 5.8% of respondents reported having "suffered a *fatal* heart attack", and 12.2% claimed to be able to recall from memory the names of every senator who has ever served in the U.S. Senate.

We created a composite variable for the highly dangerous behaviors by summing the three behaviors. Similarly, we created a composite variable for the three implausible items. Confirming our fourth hypothesis, we found a significant correlation between respondents' reported ingestion of cleaning products and other implausible behaviors (r = .44, p < .001).

Dangerous Cleaning Practices and Negative Health Outcomes

Confirming our fifth hypothesis, we found that the association between reported dangerous cleaning practices and negative health outcomes was much higher among problematic respondents compared to non-problematic respondents. Specifically, among problematic respondents, 27.2% of the variance in the number of reported health symptoms was explained by reported dangerous cleaning practices (F(1, 138) = 132.3, p < 001). In contrast, among non-problematic respondents, only 3.5% of

the variance in the number of reported health symptoms was explained by reported dangerous cleaning practices ($F(1, 458) = 17.6$, $p < 001$).

Discussion

Using data quality measures, we classified respondents as “problematic” or “non-problematic”, and we observed that the vast majority of reported dangerous cleaning practices were reported by problematic respondents. This was true across all seven dangerous behaviors, and particularly for the most dangerous (and least frequent) behaviors—ingesting a household cleaning product. Further, respondents who reported ingesting cleaning products were more likely to report other implausible behaviors. These results demonstrate that the rate of dangerous cleaning practices is largely an artifact of problematic respondent bias and that survey studies are vulnerable to such bias, particularly when attempting to detect rare events. Conclusions about the association between dangerous cleaning practices and negative health outcomes are highly dependent on the quality of the data at hand.

Though most of the reported dangerous behaviors were among problematic respondents, there was still a small minority of respondents who were not identified as problematic who did report ingesting household cleaners. While ingestion of household cleaners was reported at a very low rate among non-problematic respondents (0.9%, 0.6% and 0.5% for drinking/gargling household cleaner, soapy water and diluted bleach respectively), a key question is whether the two data quality measures used in Study 1 were sensitive enough to detect all problematic respondents.

One possibility is that even respondents who pass screens of inattentiveness and mischievousness may at times lose focus, misunderstand the intent of specific questions, or mistakenly press the wrong button when answering a question. To verify that the type of respondents who were labeled as non-problematic in Study 1 really did intentionally ingest household cleaning products to prevent a coronavirus infection, in Study 2 we added two additional instruments: response verification, and validation of demographic information.

Study 2

In Study 2 we wanted to replicate the results of Study 1 and gain further insight into the

reported dangerous cleaning behaviors among respondents who passed our data quality measures. Since the ingestion of household cleaners would be particularly alarming, we primarily focus our attention on these questions.

Hypotheses

Hypothesis 1: We hypothesized that, using the same two data quality measures, we would successfully replicate Study 1. Specifically, we predicted that 80-90% of reported ingestion household cleaning products would come from problematic respondents, and that roughly 1% of non-problematic respondents would report ingesting any one of the three cleaning products.

Hypothesis 2: We predicted that our response verification procedure would reveal that non-problematic respondents who report ingesting cleaning products either fail to confirm doing so in a follow-up question, or indicate doing so unintentionally, rather than purposely as a COVID-19 preventative measure.

Hypothesis 3: We hypothesized that an examination of respondents' open-ended responses would reveal few, if any, cogent descriptions of respondents' ingestion of cleaning products. In contrast, we expected to see informative open-ended responses of other moderately dangerous practices, such as washing produce with bleach.

Hypothesis 4: As in Study 1, we hypothesized that removing the problematic respondents and those who did not confirm having ingested household cleaner from the analytic sample would attenuate the association between dangerous cleaning practices and negative health outcomes.

Method

Participants

We collected data from a national sample of 688 respondents during the week of July 27th-July 31st, 2020. The sample was matched to the U.S. Census on gender, age, race, and region (See Table 1). As in Study 1, the InterReview IRB exempted the study from review because it is an anonymous survey. After providing informed consent, participants responded to the measures described below.

Materials

Since the survey materials were nearly identical to those employed in Study 1, we do not describe them again. We made four minor modifications to Study 2. First, instead of asking about behavior over the past month, we changed the timeframe to, "since the start of the COVID-19 pandemic in April." Second, we added response verification measures following respondents' indication of engaging in dangerous cleaning behaviors. Third, we used respondents' demographic information to further validate their responses. Fourth, we included additional exploratory measures which we do not discuss in the present report.

Response Verification

Loftus et al. showed that one way to increase accuracy in surveys responses is to ask respondents to report on behaviors of interest multiple times[50]. Their study revealed that patients tend to overreport whether they have had a physical examination, as verified by patient records[51]. They also found that incorporating a second question to verify the original response significantly improves reporting accuracy. This occurs in part because it signals to participants the importance of the question to the researcher[50].

We used an approach similar to Loftus et al., which consisted of multiple steps. First, after respondents indicated they had engaged in a dangerous cleaning behavior, they received a follow-up question asking whether they intended to respond affirmatively. For example, "You indicated you drank or gargled diluted bleach solution. Did you really drink or gargle diluted bleach solution, or did you indicate you did so by mistake on the last survey question?" Next, respondents who verified that they had indeed intended to respond affirmatively received another follow-up question to verify that the respondent had intentionally engaged in the behavior. For example, "You indicated you drank or gargled diluted bleach solution. Did you engage in this cleaning behavior intentionally?" Finally, we asked all respondents who reported engaging in a dangerous cleaning behavior to provide more context about their answer in an open-ended format: "Please describe the steps you took to clean this

way. What cleaning product did you use? How did you administer it? This research is very important for public health policy and we very much appreciate your time and input!"

Demographic Verification

The final method we use to identify problematic respondents is based on Robinson-Cimpians' method of validating demographic information[16]. This method involves looking across reported demographics to find inconsistencies and exaggerated claims. Robinson-Cimpian developed a quantifiable metric for flagging problematic respondents based on an outlier analysis. Here, we utilize a similar approach by flagging demographic claims that are clearly implausible. Among respondents who passed all previous data quality measures (i.e., found to be attentive, not mischievous, and verified that they had intentionally engaged in dangerous cleaning practices as a preventative COVID-19 measure), we examined their reported age, height, weight, and parental status, looking for extreme outliers and implausible entries.

Results

As in Study 1, we first classified respondents into one of two groups: "problematic respondents" were those who responded incorrectly to the attentiveness and mischievousness measures, and "non-problematic" respondents were those who responded correctly to the attentiveness and mischievousness measures. Based on these measures, which were identical to the data quality measures in Study 1, 473 respondents (68.8%) were "non-problematic," and 215 respondents (31.2%) were problematic.

Study 1 Replication

Before investigating the results of the response verification and demographic verification we first wanted to determine whether our results were similar to those we obtained in Study 1, specifically with respect to the ingestion of household cleaning products (cleaner, soap, or diluted bleach). In the full sample, 55 respondents (8%) reported ingesting at least one of these substances However, as in Study 1, we again found that most (43 of 55, or 78%) of these reports were made by problematic respondents. Among non-problematic respondents, reports of ingesting household

cleaner, soapy water, or diluted bleach were 1.05%, 1.48% and 0.63% respectively. In total, 2.54% of non-problematic respondents reported ingesting at least one of the products. Thus, we confirmed H1—most reported ingestions of household cleaning products came from problematic respondents, and very few non-problematic respondents (12) reported ingesting any household cleaning products.

Response Verification

Our second hypothesis was that the response verification method would reveal that most respondents who reported ingesting cleaning products would either fail to confirm doing so or would indicate that they did so unintentionally. As mentioned above, only 12 of the 473 respondents passed the first two data quality measures and reported ingesting at least one cleaning product. The first response verification question asked participants if they had intentionally selected “yes” to ingesting a cleaning product. Of the 12 non-problematic respondents who reported ingesting a cleaning product, only 3 (0.63% of non-problematic respondents) confirmed that they had indeed intended to select “yes” to the question about ingesting a cleaning product.

The second response verification question asked respondents whether they had intentionally ingested the cleaning product. Of the 3 remaining respondents, 2 reported unintentionally ingesting a cleaning product (and therefore not having done so to avoid COVID-19). These results confirm H2—among non-problematic respondents, only 1 respondent both verified that they had ingested a cleaning product and that they had intentionally done so. See Figure 3 for a flow chart of the verification process.

Demographic Verification

Next, we examined the pattern of demographic information provided by this one respondent. Several elements of their demographic data were suspicious. First, this participant reported being 20 years old and having four children. Having four children is certainly not uncommon but having four children at 20 years old is unusual, especially in the US. This respondent also reported a weight of 1900 pounds, and a height of “100”. Although they did not indicate whether they responded in centimeters or inches, either (8.3 feet or 3.2 feet) is an extreme outlier.

FIGURE 3. Flow chart of the step-wise process of verifying the ingestion of cleaning products

Open-Ended Responses

After excluding respondents with bad data quality (N = 43), and those who did not verify their response (N = 11), only one respondent remained. This respondent provided highly suspicious demographic information (see above), but we examined their open-ended responses to gain further clarity. When asked to provide more detail about having ingested cleaning products, this respondent's response was "YXgyvuguhih".

Across the entire sample, the open-ended responses further indicated that at least some respondents misunderstood the questions. For example, one respondent who reported drinking and gargling soapy water indicated on the open-ended response that, "My mother made me wash my mouth out with soap water because I was cursing, and I accidentally swallowed some". It is clear from this response that the respondent did not read the question carefully and did not consider that (a) the question specifically inquired about practices in the time of Covid-19, and (b) specifically inquired about cleanliness practices that were motivated by reducing the likelihood of Covid-19 infection, rather than something that happened to them in childhood. This specific respondent was also flagged by the attentiveness instrument. Their open-ended response provides corroborating support for flagging this respondent as problematic by the attentiveness instrument, and further demonstrates how answers to Yes/No questions cannot always be taken at face value.

Overall, we did not find a single respondent who provided any reasonable or compelling open-ended descriptions of ingestion of cleaning products. This is in contrast with other cleaning practices reported on the survey. For example, regarding "misting the body with cleaning spray or alcohol spray after being in public spaces", the open-ended responses were clear and detailed, e.g. "I use 70% alcohol in a spray mist bottle when I take my clothes off and I mist them, let them air dry and then put them in the washing machine." In general, the open-ended responses indicate that many of the

questions were at times misinterpreted even by non-problematic participants. This raises the possibility that the estimates obtained for the non-ingestion practices are also inflated among non-problematic respondents. These results confirm H3: there were no cogent responses indicating ingestion of cleaning products to prevent a COVID-19 infection, whereas there were clear and informative reports of other less dangerous cleaning practices.

Dangerous Cleaning Practices and Negative Health Outcomes

Confirming our fourth hypothesis, we found that reported dangerous cleaning practices accounted for 14.7% of the variance in the number of reported health symptoms among respondents who did not pass the screener or did not verify ingesting cleaners, $F(1, 213) = 36.7$, $p < 001$. However, among non-problematic respondents who verified their responses this association was not significant. Specifically, less than .001% of the variance in the number of reported health symptoms was explained by reported dangerous cleaning practices, $F(1, 398) = 0.556$, $p = .82$.

Discussion

In Study 2, we replicated the results of Study 1, showing that most respondents who reported engaging in highly dangerous cleaning practices were inattentive or mischievous. Further, we employed a response verification approach for the remaining respondents who reported engaging in dangerous cleaning practices, and found that 100% of these reports can be explained by unintentionally selecting “yes,” misreading the question, or having low quality open-ended responses and inconsistent demographic information.

General Discussion

The goal of the present study was to examine whether claims that respondents make about engaging in dangerous cleaning practices to protect themselves against Covid-19 are in large part an artifact of problematic respondent bias. Across two studies, with close to 1300 total respondents, we replicated the CDC’s findings[22] showing that around 4% of respondents reported engaging in each of the three highly dangerous behaviors we asked about in the survey: drinking or gargling household cleaner, soapy water, and diluted bleach. However, consistent with the notion that problematic

respondents can create the illusion that almost anything occurs in the population no matter how implausible, we also observed that 3-7% of respondents reported having never used the Internet (an answer they provided while using the internet), and having suffered a *fatal* heart attack. These findings are consistent with a recent comprehensive report by the Pew Research Center that 7% of respondents from over 50 different opt-in panels provide “bogus” data[23], as well as the “lizardman’s constant” argument[52], that approximately 4% of survey respondents can be expected to provide nonsense responses to any question.

After having categorized respondents into problematic and non-problematic groups based on inattention and acquiescence, we observed that all reports of highly dangerous cleaning practices were made by problematic respondents. Once inattentive, acquiescent, and careless responses were removed from the analytic sample, we find no evidence that people intentionally ingest household cleaning products for protection against Covid-19.

Types of problematic respondent bias and their effects

We observed that problematic respondent bias introduces two sources of error; 1) random responding increases noise and 2) acquiescence introduces systematic bias.

Random Responding

Some problematic respondents tend to randomly select from among the available response options. This decreases the signal-to-noise ratio, tending to drive estimates toward the mean of the distribution. Random responding not only makes rare practices appear more common than they actually are, but also makes more common practices appear less common than they actually are. Specifically, the majority of non-problematic respondents reported having increased general non-dangerous cleaning practices to prevent Covid-19 infection. However, problematic respondents were less likely to report engaging in general non-dangerous cleaning practices than non-problematic respondents. This finding is consistent with the idea that additional noise attenuates estimates toward the middle of the distribution.

We also found that bias is proportionally greater among the lowest frequency events.

Specifically, we observed that problematic respondents were three times more likely to report using cleaning products on hands or bare skin compared to non-problematic respondents. However, problematic respondents were twenty-nine times more likely to report gargling or drinking bleach solution compared to non-problematic respondents. Across all cleaning practices examined in this study, the more common practices were proportionally less likely to be affected by problematic respondent bias.

Acquiescence Bias

Some problematic respondents systematically select a "Yes" response from among the available response options. While random responding introduces noise, systematic yea-saying introduces error that is correlated across unrelated items. Evidence of systematic yea-saying among problematic respondents can be seen by examining the correlation between cleaning practices and implausible behaviors, as well as between cleaning practiced and negative health outcomes. Among problematic respondents, over 25% of variance in health outcomes is explained by dangerous cleaning practices. However, among non-problematic respondents this relationship was not significant. This shows that problematic respondents systematically answer *yes* to a variety of questions across the survey, artificially driving up associations between unrelated events. These results are consistent with previous studies showing that associations between variables are dramatically reduced or eliminated altogether when problematic respondents are removed from a sample[16].

Which dangerous cleanliness practices are people actually engaging in?

The spread of COVID-19 across the US and the world created fear of contagion, leading people to seek ways of protecting themselves against infection. The current study explored which specific cleanliness practices people started to engage in during the COVID-19 pandemic, querying respondents about seven practices that are considered dangerous and are not recommended by the CDC. Overall, we find that the reported rates of all seven cleanliness practices examined in this study are dramatically lower than previously thought because most reports of these practices are provided by problematic respondents. The three practices that involve ingesting household cleaning products, in

particular, are entirely an artifact of problematic respondent bias, misreading questions, and respondent error. At the same time, more than 5% of non-problematic respondents reported engaging in several dangerous cleaning practices, including washing food products with bleach, using cleaner or disinfectant on hands and bare skin, and misting the body with cleaning products. What can be concluded about the rates at which people engage in these practices, and what are the implications of these findings for public health?

Overall, the interpretation of reports of non-ingestion related dangerous cleaning practices is complicated by open-ended responses that make it clear that some non-problematic respondents did not fully understand the intent of these questions. As is evident from open-ended responses, even otherwise attentive and well-intentioned survey-takers can misunderstand the intention of questions or may not be perfectly aware of the meaning of specific terms and phrases. For example, the meaning of the term "household cleaner" was not clear to all respondents, and at least some put regular soap in that category. To the question "Did you use household cleaner to clean or disinfect bare hands or skin?", examples of open-ended responses included: "I washed my hands with antibacterial soap". The use of antibacterial soap is not considered a dangerous cleaning practice. Thus, the interpretation of reports of using household cleaner on bare skin hinges on whether people categorized various non-dangerous cleaners such as soap as a "household cleaner".

For inhaling vapors of household cleaners, there was also evidence of misinterpretation. Vapors from a wide variety of household products, including cleaners, can be inhaled. The question in the CDC study[22] was specifically intended to detect whether people are inhaling cleaners for the purpose of preventing infection. To achieve this goal, vapors of household cleaner would presumably be inhaled by breathing directly from an open-air container, but the current question does not specify the specific method of delivery. This led some respondents to interpret the question to mean any inhalation of cleaner, even if that cleaner was inhaled during a normal cleaning routine. For example, in response to the question "Did you inhale the vapors of household cleaners like bleach?" examples of open-ended responses included: "I poured product on the floor and began to mop". While inhaling

vapors of cleaning products during daily cleaning activities such as mopping can have negative long-term health consequences, such cleaning practices are not considered to pose an acute danger to health.

Other questions also showed evidence of misinterpretation. To the question "Did you mist the body with cleaning spray or alcohol spray after being in public spaces?", examples of open-ended responses included: "I used 70% alcohol in a spray mist bottle when I take my clothes off and I mist them, let them air dry and then put them in the washing machine". This respondent did mist with alcohol, but did not do so directly on the body, and then washed off the alcohol in the washing machine before their clothes made direct contact with the body. To the question of "Why did you wash fruits, vegetables, or other food products with bleach?" examples of misinterpretation included "I used soap and a special sponge to make sure I get a deep clean on the food I serve to my son". As many open-ended responses show, people often generalize to cleaning products other than the specific one that is mentioned in the question.

Overall, for all questions, open-ended responses make it evident that the practices that at least some people were reporting on are not considered dangerous. These responses show that at least some people did not realize the importance of bleach as a specific cleaner of interest of several questions and were reporting on using cleaners like soap on their hands and skin or to clean fruits and vegetables. On other questions, respondents were affirming that they engaged in a certain practice, like misting alcohol, but did not realize the importance of other parts of the question, which focused on direct contact with the skin. Additionally, respondents did not always make a distinction between practices that they were engaged in prior to the start of the pandemic and those that they started doing specifically to prevent Covid-19 infection.

Given the uncertainties in the way that these questions were understood by respondents, the implications of these reported practices for public health remain uncertain. And it is not clear from these findings whether substantial numbers of people engage in specific dangerous practices. To fully understand the implication of these cleaning practices for public health, future studies should examine

these practices in more detail, focusing on several details not addressed here or in previous studies: 1) effort should be made to define the specific activities and substances in the question so as to make it very clear to respondents which activities the researchers are asking about, and 2) efforts should be made to specifically define terms such as “household cleaner” so as to leave no room for doubt that the practices in question pose a health risk. Because the current study and previous studies that used these questions were not designed to provide a systematic examination of any of these practices, it remains difficult to ascertain whether the practices reported on in this survey were being practiced at all and whether they pose a substantive risk to public health.

Public health implications

While there is some evidence of an increase in dangerous practices in the reported rise in calls to poison control centers at the start of the pandemic[53], it is unclear if the greater volume of calls was due to intentional or unintentional ingestion/inhalation of cleaning products. It is likely that due to the increase in overall frequency of regular cleaning practices, accidental exposure to, and ingestion of, dangerous chemicals may have occurred more frequently. The likelihood that the majority of such reports were accidental or not related to Covid-19 concerns is corroborated by several observations. First, half of the calls to poison centers were for children under age 5. Furthermore, while there were 28,158 calls to poison control centers related to disinfectants in January-March of 2020, there were 25,021 such calls during the same period in 2019. Thus, there is a very high base rate of contamination incidents stemming from improper or accidental use of disinfectants that have no connection to Covid-19. The high base rate of such incidents is attributable to multiple factors, the largest of which is accidental contamination by children and adults. But other likely factors include the use of industrial bleaches such as chlorine dioxide by people who falsely believe in their general medicinal properties. Such beliefs are most prevalent in Latin America, particularly among certain groups of individuals, including physicians, who favor homeopathic and non-traditional medicine[54]. Such practices likely also exist in United States, but less is known about their prevalence there. While the use of industrial bleach for medical purposes is widespread among some groups, it precedes

Covid-19 by decades.

Additionally, several practices examined in this study, including the ingestion and inhalation of household cleaner, have been documented in the medical literature[55]. Such practices are most commonly observed among vulnerable populations, such as low socioeconomic groups, teenagers, and prison inmates. There have been several documented cases of blindness and death due to drinking hand sanitizer since the start of the Covid-19 pandemic. However, in virtually all cases, people ingest and inhale sanitizer for its alcohol content and psychotropic effects[56]. It is thus possible that the use of sanitizer for its psychotropic effects increased at the start of Covid-19 due to the psychological toll that the pandemic had, particularly on the most vulnerable groups.

Overall, among all 55 poison control call centers in the United States, reported disinfectants-related incidents among adults increased by a few thousand at most during the first three months of COVID compared to the same time in previous years[53]. While this increase is significant, it constitutes less than 1/1000th of one percent of the US population. The specific reasons for this increase are not known. However, even if some of these cases can be attributed to the intentional ingestion of bleach and disinfectants to prevent Covid-19 infection, it is unlikely to account for the 4% of ingestion of household cleaner reported in the CDC study[22]. Instead, as we show, that 4% ingestion rate is fully accounted for by problematic respondent bias. Overall, the data presented in our study do not contradict that some individuals may have ingested bleach to prevent Covid-19 infection. Our data do show, however, that such practices are so infrequent so as not to be detectable on national surveys.

Health behavior theories emphasize that social norms can impact individual decision making[57] and may be even more salient among vulnerable populations. Experimental evidence suggests that social norms can alter risk perception, thereby influencing behavioral choices[58], and increasing vulnerability to misinformation[59]. Presenting practices such as the ingestion and inhalation of household cleaning products as being practiced by tens of millions of people risks normalizing such practices and potentially inadvertently reinforcing them. For this reason, presenting the results of surveys that are subject to problematic respondent bias is itself a matter of public health concern. The

reporting of any rare event detected on a survey should be subjected to rigorous examination and should require an additional level of stringency when screening respondents.

Recommendations for Eliminating Problematic Respondents

Detecting rare events requires an additional level of stringency when screening respondents. What is generally sufficient for most studies may prove inadequate when looking at low frequency events. When survey data suggest that people are engaging in surprising and extremely unusual behaviors—especially those with important public health implications—it is critical to examine whether such results may have been influenced by problematic respondents prior to drawing strong conclusions from the data. Here, we recommend specific practices that researchers should follow when collecting data online. These practices will improve data quality on surveys seeking to measure rare events as well as improve the signal to noise ratio in any survey.

Do not rely on third-party solutions without testing them first.

Standard procedures used in the opt-in panel industry to protect surveys against bad data do not confer sufficient protection for the vast majority of scientific surveys. This is especially true when the goal is to detect relatively rare events. No solution is perfect, and even if a solution works to protect certain types of surveys there is no guarantee that it will work in all cases. For example, some data quality solutions employed by opt-in panels may protect against duplicate responses, bots, straight lining, and virtual private networks (VPNs) which conceal a respondent’s country of origin, but may not be effective against inattentiveness, mischievous respondents, or acquiescence bias.

Use response validation.

It is important to follow up with individual respondents to gather more detailed information about reported behaviors, especially in the case of rare events. Researchers can ask respondents to describe these practices in an open-ended format, such as to provide specific examples of their behavior, to provide more context, and to describe the rationale for their practices. Researchers could even set up video interviews with select respondents to verify that the respondents are indeed real, that they fully understand what is being asked of them, and that their behaviors are being reported

accurately. Even a handful of such interviews can provide important evidence that such practices are really occurring in the population.

Use validated instruments.

Validated screeners and other forms of intra and extra-survey data quality measures should be incorporated into all surveys as protection against problematic respondents. Throughout this paper we have described a variety of screening mechanisms that have been developed and validated by multiple research teams. Other more in-depth discussions of how to develop and incorporate data protection measures have been described elsewhere. We consider a data quality measure to be validated when it accomplishes at least the following three goals: (1) it has been tested in a sample of attentive participants (as measured through previously validated measures), and the vast majority of these participants answered correctly, (2) it specifically detects inattention or acquiescence, rather than participants' memory capacity, education level, or cultural knowledge, and (3) it is neither overly stringent nor overly lenient. This last requirement typically requires extensive testing.

Conclusion

We found that dangerous cleaning practices were exclusively reported by problematic respondents, indicating that reports of high-risk cleaning practices to provide protection against Covid-19 were an artifact of problematic respondent bias. Failing to screen problematic respondents causes rare events to be severely overestimated and normal practices to be underestimated. Over the last several decades our society has become increasingly dependent on survey research, with more than 80% of surveys using online respondents for at least some of the data collection (Kennedy et al., 2020). Problematic survey respondents pose a fundamental challenge to all survey research and threaten the validity of public-health policy. For this reason, it is critical to develop validated instruments to protect surveys against such bias. To mitigate against these threats, researchers should rigorously check for problematic respondents, particularly when the survey aims to measure rare events. Using these techniques significantly increases the accuracy of measurement and prevents problematic respondents from invalidating survey results.

References

1. Singleton R, Straits BC. *Approaches to social research*. Oxford University Press.doi:10.1007/s11205-015-1002-x; 209AD.
2. Given L. Survey Research. In: *The SAGE Encyclopedia of Qualitative Research Methods*. ; 2008. doi:10.4135/9781412963909.n441
3. Connelly R, Gayle V, Lambert PS. A Review of occupation-based social classifications for social survey research. *Methodol Innov*. 2016;9:205979911663800. doi:10.1177/2059799116638003
4. Groves RM, Fowler Jr FJ, Couper MP, Lepkowski JM, Singer E, Tourangeau R. *Survey Methodology*. John Wiley & Sons; 2011.
5. Saris WE, Gallhofer IN. *Design, Evaluation, and Analysis of Questionnaires for Survey Research*. John Wiley & Sons; 2014.
6. Wolf C, Joye D, Smith TW, Fu Y. *The SAGE Handbook of Survey Methodology*. SAGE Publications; 2016.
7. Mutz DC, Kim E. The Progress and Pitfalls of Using Survey Experiments in Political Science. *Oxf Res Encycl Polit*. Published online 2020. doi:10.1093/acrefore/9780190228637.013.929
8. Ezzati-Rice TM, Curtin LR. Population-based surveys and their role in public health. *Am J Prev Med*. 2001;20(4):15-16.
9. Belisario JS, Jamsek J, Huckvale K, O'donoghue J, Morrison CP, Car J. Comparison of self-administered survey questionnaire responses collected using mobile apps versus other methods. *Cochrane Database Syst Rev*. Published online 2015.
10. Saczynski JS, Mcmanus DD, Goldberg RJ. Commonly Used Data- collection Approaches in Clinical Research. *Am J Med*. 2013;126(11):946-950. doi:10.1016/j.amjmed.2013.04.016
11. Safdar N, Abbo LM, Knobloch MJ, Seo SK. Research Methods in Healthcare Epidemiology: Survey and Qualitative Research. *Infect Control Hosp Epidemiol*. 2017;37(11):1272-1277. doi:10.1017/ice.2016.171
12. Kim DS, McCabe CJ, Yamasaki BL, Louie KA, King KM. Detecting random responders with infrequency scales using an error-balancing threshold. *Behav Res Methods*. 2018;50(5).
13. King KM, Kim DS, McCabe CJ. Random responses inflate statistical estimates in heavily skewed addictions data. *Drug Alcohol Depend*. 2018;183:102-110.
14. Chandler J, Paolacci G, Hauser DJ. Data quality on Mechanical Turk. Litman L, Robinson J, eds. *Conduct Online Res Amaz Mech Turk Beyond*. 2020;1.
15. Fan X, Miller BC, Park KE, et al. An Exploratory Study about Inaccuracy and Invalidity in Adolescent Self-Report Surveys. *Field Methods*. 2006;18(3):223-244. doi:10.1177/152822X06289161
16. Robinson-Cimpian JP. Inaccurate estimation of disparities due to mischievous responders: Several suggestions to assess conclusions. *Educ Res*. 2014;43(4):171-185.
17. Kramer J, Rubin A, Coster W. Strategies to address participant misrepresentation for eligibility in Web-based research. *Int J Methods Psychiatr Res*. 2014;23(1):120-129.
18. Fish JN, Russell ST. Have mischievous responders misidentified sexual minority youth disparities in the National Longitudinal Study of Adolescent to Adult Health? *Arch Sex Behav*. 2018;47(4):1053-1067.
19. Kaltiala-Heino R, Lindberg N. Gender identities in adolescent population: methodological issues and prevalence across age groups. *Eur Psychiatry*. 2019;55:61-66.
20. Li CR, Follingstad DR, Campe MI, Chahal JK. Identifying invalid responders in a campus climate survey: types, impact on data, and best indicators. *J Interpers Violence*. 2020;0886260520918588.
21. Cimpian JR, Timmer JD. Mischievous responders and sexual minority youth survey data: a brief history, recent methodological advances, and implications for research and practice. In: *Archives of Sexual Behavior*. ; 2020:1-6.
22. Gharpure R, Hunter CM, Schnall AH, et al. Knowledge and practices regarding safe household cleaning and disinfection for COVID_J19 prevention—United States. *Morb Mortal Wkly Rep*. 2020;69.
23. Kennedy C, Hately N, Lau A, et al. Assessing the risks to online polls from bogus respondents.

Pew Res Cent. Published online 2020. https://www.pewresearch.org/methods/2020/02/18/assessing-the-risks-to-
24. Petzel TP, Johnson JE, McKillip J. Response bias in drug surveys. *J Consult Clin Psychol*. 1973;40(3):437-439. doi:10.1037/h0034439
25. Pape H, Storvoll EE. Teenagers' "use" of Non-Existent Drugs: A study of false positives. *Nord Stud Alcohol Drugs*. 2006;23(1_suppl):31-46.
26. Chandler J, Rosenzweig C, Moss AJ, Robinson J, Litman L. Online panels in social science research: Expanding sampling methods beyond Mechanical Turk. *Behav Res Methods*. 2019;51(5):2022-2038.
27. Cornell D, Klein J, Konold T, Huang F. Effects of validity screening items on adolescent survey data. *Psychol Assess*. 2012;24(1):21.
28. Ward MK, Pond SB. Using virtual presence and survey instructions to minimize careless responding on Internet-based surveys. *Comput Hum Behav*. 2015;48:554-568. doi:10.1016/j.chb.2015.01.070
29. Litman L, Robinson J, Rosenzweig C. The relationship between motivation, monetary compensation, and data quality among US- and India-based workers on Mechanical Turk. *Behav Res Methods*. 2015;47(2):519-528. doi:10.3758/s13428-014-0483-x
30. Shukla K, Konold T. A two-step latent profile method for identifying invalid respondents in self-reported survey data. *J Exp Educ*. 2018;86(3):473-488.
31. Steedle JT, Hong M, Cheng Y. The Effects of Inattentive Responding on Construct Validity Evidence When Measuring Social–Emotional Learning Competencies. *Educ Meas Issues Pract*. 2019;38(2):101-111.
32. Leiner DJ. Too Fast, too Straight, too Weird: Non-Reactive Indicators for Meaningless Data in Internet Surveys. *Surv Res Methods*. 2019;13(3):229-248. doi:10.18148/srm/2019.v13i3.7403
33. Cimpian JR, Timmer JD. Large-scale estimates of LGBQ-heterosexual disparities in the presence of potentially mischievous responders: A preregistered replication and comparison of methods. *AERA Open*. 2019;5(4).
34. Fan X, Miller BC, Christensen M, et al. Questionnaire and interview inconsistencies exaggerated differences between adopted and non-adopted adolescents in a national sample. *Adopt Q*. 2002;6(2):7-27.
35. Vriesema CC, Gehlbach H. *Assessing Survey Satisficing: The Impact of Unmotivated Questionnaire Respondents on Data Quality*. Working Paper. Policy Analysis for California Education; 2019.
36. Jia Y, Konold TR, Cornell D, Huang F. The impact of validity screening on associations between self-reports of bullying victimization and student outcomes. *Educ Psychol Meas*. 2018;78(1):80-102.
37. Litman L, Robinson J. *Conducting Online Research on Amazon Mechanical Turk and Beyond*. SAGE Publications; 2020.
38. Downes-Le Guin T, Mechling J, Baker R. Great results from ambiguous sources: Cleaning Internet panel data. In: *ESOMAR World Research Conference: Panel Research*. ; 2006.
39. Courtright M, Miller C. Respondent Validation: So many choices. Does choice of vendor make a difference in respondent validation and how does it impact the overall sampling frame and data quality. In: *CASRO Online Research Conference*. ; 2011.
40. Chakraborty N. Online MR Quality: Is ignorance bliss? *MRA's Alert Mag*. Published online 2014:12-16.
41. Hays RD, Liu H, Kapteyn A. Use of Internet panels to conduct surveys. *Behav Res Methods*. 2015;47(3):685-690. doi:10.3758/s13428-015-0617-9
42. Teitcher JEF, Bockting WO, Bauermeister JA, Hoefer CJ, Miner MH, Klitzman RL. Detecting, Preventing, and Responding to "Fraudsters" in Internet Research: Ethics and Tradeoffs. *J Law Med Ethics*. 2015;43(1):116-133. doi:10.1111/jlme.12200
43. Kees J, Berry C, Burton S, Sheehan K. An Analysis of Data Quality: Professional Panels,

Student Subject Pools, and Amazon's Mechanical Turk. *J Advert*. 2017;46(1):141-155.
44. Litman L, Robinson J, Rosenzweig C. Data representativeness in online samples. Litman L, Robinson J, eds. *Conduct Online Res Amaz Mech Turk Beyond*. 2020;1.
45. CDC. Community, Work, and School. Centers for Disease Control and Prevention. Published February 11, 2020. Accessed June 15, 2022. https://www.cdc.gov/coronavirus/2019-ncov/community/clean-disinfect/index.html
46. World Health Organization. Coronavirus disease (COVID-19): Cleaning and disinfecting surfaces in non-health care settings. Accessed June 15, 2022. https://www.who.int/news-room/questions-and-answers/item/coronavirus-disease-covid-19-cleaning-and-disinfecting-surfaces-in-non-health-care-settings
47. Litman L, Williams MT, Rosen Z, Weinberger-Litman SL, Robinson J. Racial disparities in cleanliness attitudes mediate purchasing attitudes toward cleaning products: A serial mediation model. *J Racial Ethn Health Disparities*. 2018;5(4):838-846.
48. Litman L, Robinson J, Weinberger-Litman SL, Finkelstein R. Both intrinsic and extrinsic religious orientation are positively associated with attitudes toward cleanliness: Exploring multiple routes from godliness to cleanliness. *J Relig Health*. 2019;58(1):41-52.
49. Larson E. Hygiene of the skin: when is clean too clean? *Emerg Infect Dis*. 2001;7(2):225.
50. Loftus EF, Klinger MR, Smith KD, Fiedler J. A tale of two questions: Benefits of asking more than one question. *Public Opin Q*. 1990;54(3):330-345.
51. Armstrong K, Long JA, Shea JA. Measuring adherence to mammography screening recommendations among low-income women. *Prev Med*. 2004;38(6).
52. Alexander S. Lizardman's Constant Is 4%. Slate Star Codex. Published April 12, 2013. Accessed June 15, 2022. https://slatestarcodex.com/2013/04/12/noisy-poll-results-and-reptilian-muslim-climatologists-from-mars/
53. Chang A. Cleaning and Disinfectant Chemical Exposures and Temporal Associations with COVID-19 — National Poison Data System, United States, January 1, 2020–March 31, 2020. *MMWR Morb Mortal Wkly Rep*. 2020;69. doi:10.15585/mmwr.mm6916e1
54. Mostajo-Radji MA. Pseudoscience in the times of crisis: how and why chlorine dioxide consumption became popular in Latin America during the COVID-19 pandemic. *Front Polit Sci*. 2021;3:25.
55. Williams H, Moyns E, Bateman DN, Thomas SH, Thompson JP, Vale JA. Hazard of household cleaning products: a study undertaken by the UK National Poisons Information Service. *Clin Toxicol*. 2012;50(8):770-775.
56. Rayar P, Ratnapalan S. Pediatric ingestions of house hold products containing ethanol: a review. *Clin Pediatr (Phila)*. 2013;52(3):203-209.
57. Legros S, Cislaghi B. Mapping the social-norms literature: An overview of reviews. *Perspect Psychol Sci*. 2020;15(1):62-80.
58. Veflen N, Scholderer J, Langsrud S. Situated food safety risk and the influence of social norms. *Risk Anal*. 2020;40(5):1092-1110.
59. Myrick JG, Erlichman S. How audience involvement and social norms foster vulnerability to celebrity-based dietary misinformation. *Psychol Pop Media*. 2020;9(3):367.

Figure 1

Click here to access/download;Figure;Figure1.png

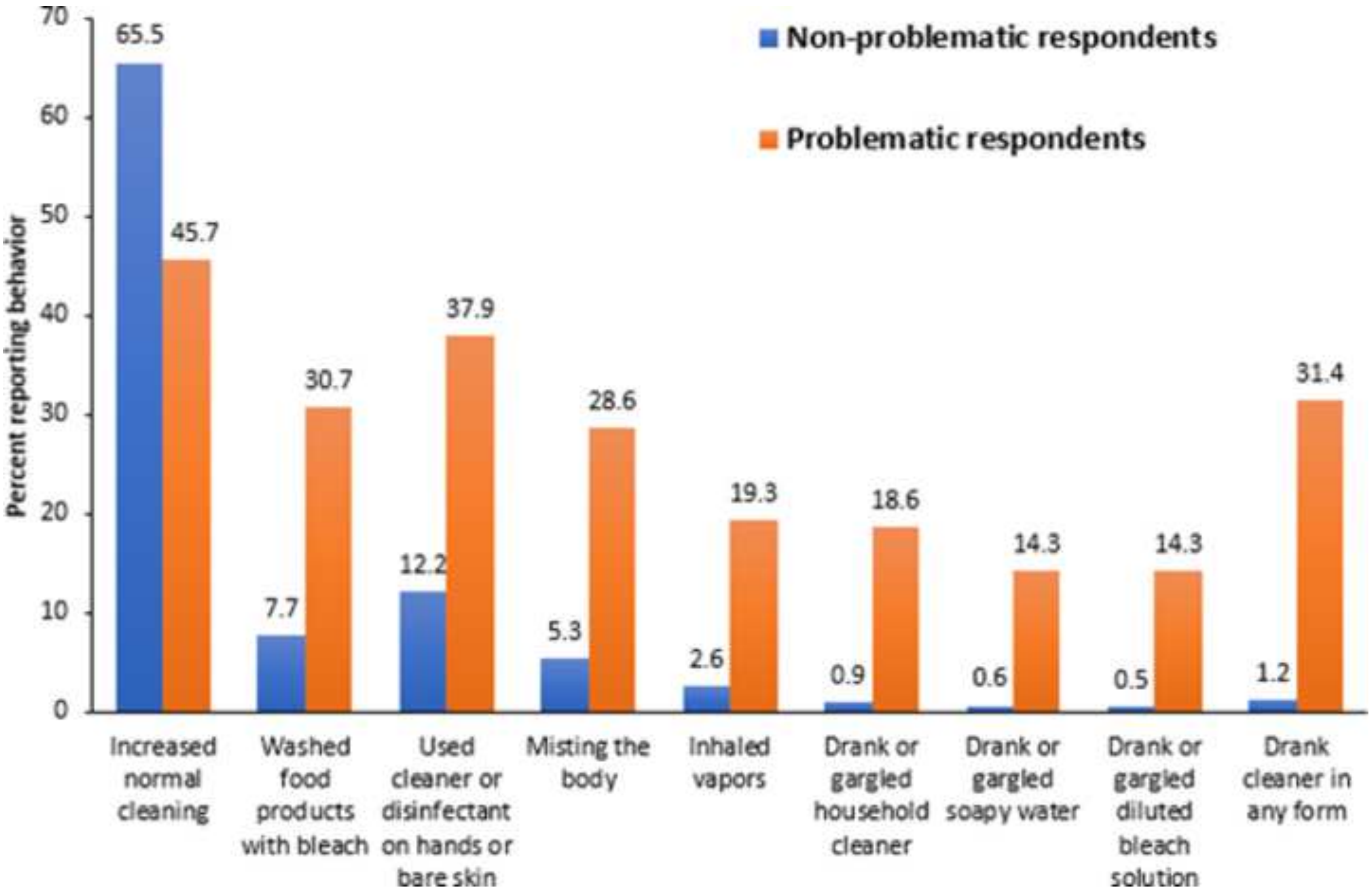

Figure 2

Click here to access/download;Figure;Figure2.png

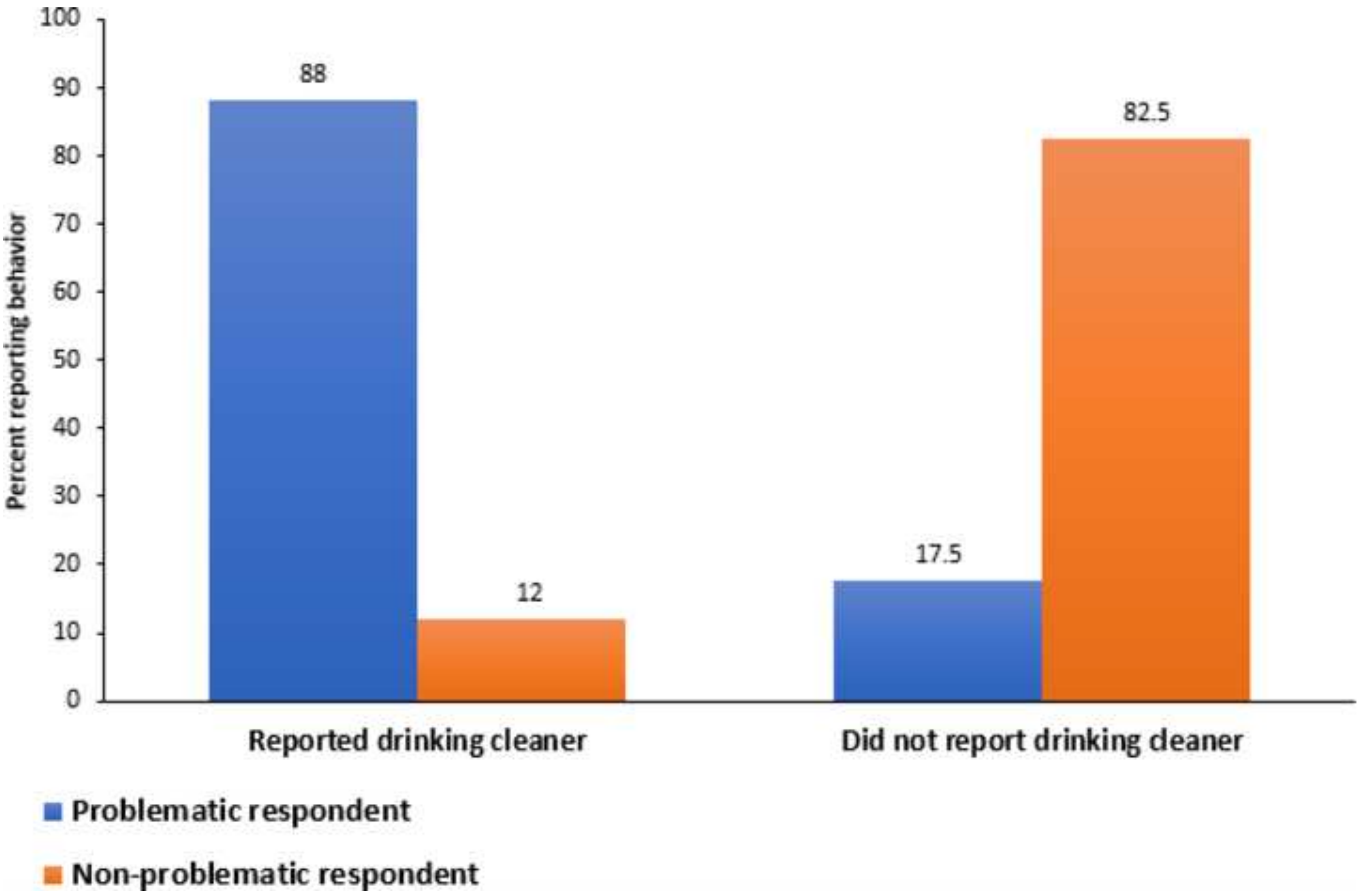

Figure 3 Click here to access/download;Figure;Figure3.jpg

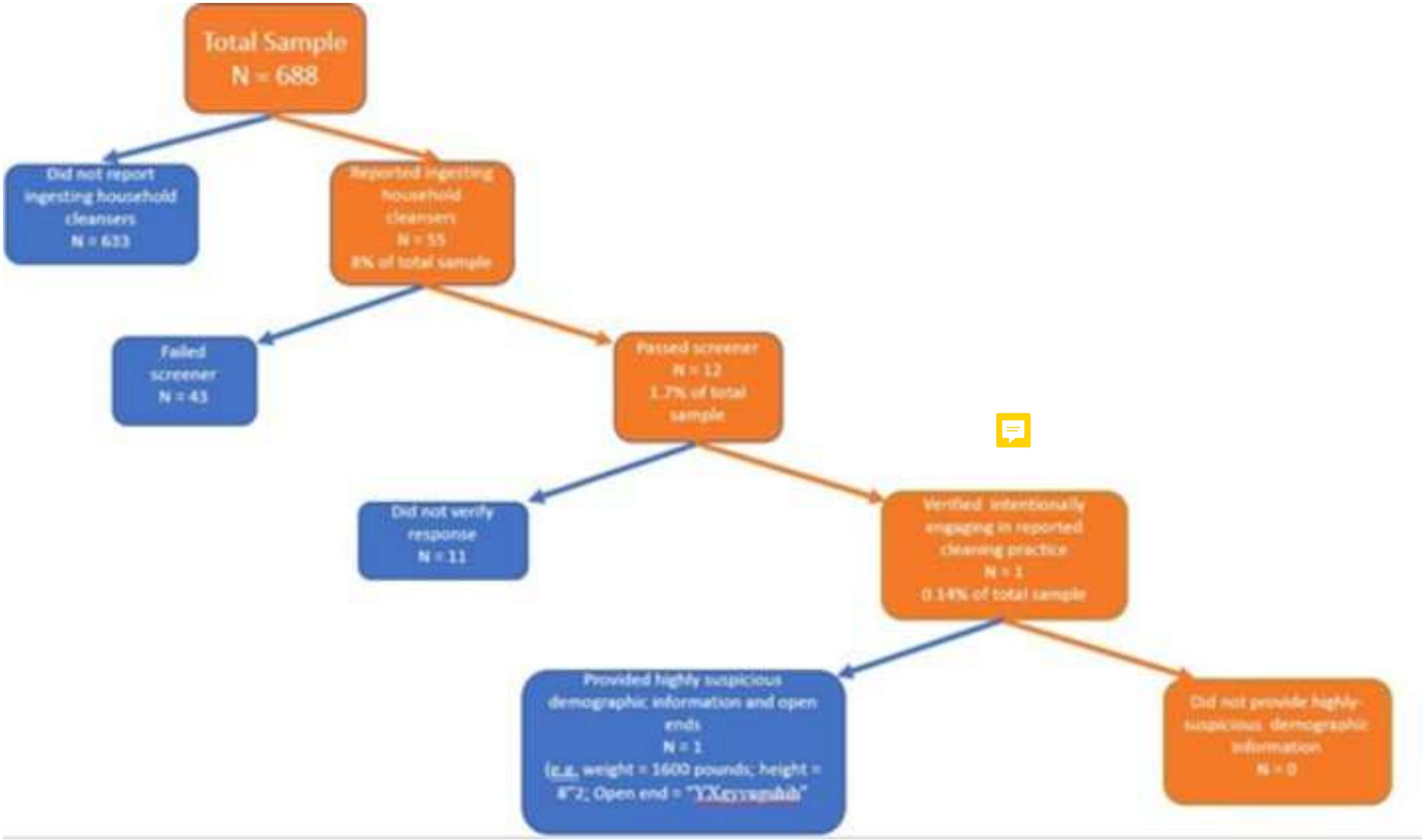

Supplement: Attachment — Submitted filename: PONE-D-22-20912_reviewer_comments.pdf [file pone.0287837.s001.pdf]
